# Supplementary material for: Meta-Analysis and Gene Set Enrichment Relative to ER Status Reveal Elevated Activity of MYC and E2F in the “Basal” Breast Cancer Subgroup
Source: PLoS One. 2009 Mar 9;4(3):e4710. doi: 10.1371/journal.pone.0004710 (PMC2650420; doi:10.1371/journal.pone.0004710)
Supplement: Dataset S1 — GSEA results from first screen. The file contains a zipped archive of the HTML results of the second GSEA screen. Reports for enriched or depleted datasets may be accessed from the “index.html” link within the my_analysis.GseaPreranked.1219975950625.rpt.zip ZIP archive. Enrichment plots are available for each of the 50 most enriched and depleted gene sets. (13.32 MB ZIP) [file pone.0004710.s001.zip › my_analysis.GseaPreranked.1219975950625.rpt.zip!//ZHAN_MULTIPLE_MYELOMA_SUBCLASSES_DIFF.html]

Details for gene set ZHAN\_MULTIPLE\_MYELOMA\_SUBCLASSES\_DIFF[GSEA]

|  || Dataset | zSco.ERneg.to.ERpos\_collapsed |
| Phenotype | NoPhenotypeAvailable |
| Upregulated in class | na\_pos |
| GeneSet | ZHAN\_MULTIPLE\_MYELOMA\_SUBCLASSES\_DIFF |
| Enrichment Score (ES) | 0.6784692 |
| Normalized Enrichment Score (NES) | 2.2202232 |
| Nominal p-value | 0.0 |
| FDR q-value | 0.0 |
| FWER p-Value | 0.0 |
Table: GSEA Results Summary

  

Fig 1: Enrichment plot: ZHAN\_MULTIPLE\_MYELOMA\_SUBCLASSES\_DIFF      
 Profile of the Running ES Score & Positions of GeneSet Members on the Rank Ordered List

  

| PROBE | GENE SYMBOL | GENE\_TITLE | RANK IN GENE LIST | RANK METRIC SCORE | RUNNING ES | CORE ENRICHMENT || 1 | EZH2 |  |  | 312 | 4.888 | 0.0433 | Yes |
| 2 | RANBP1 |  |  | 380 | 4.733 | 0.1029 | Yes |
| 3 | CKS1B |  |  | 416 | 4.638 | 0.1636 | Yes |
| 4 | XRCC6 |  |  | 581 | 4.186 | 0.2084 | Yes |
| 5 | MSH2 |  |  | 819 | 3.748 | 0.2417 | Yes |
| 6 | UGP2 |  |  | 822 | 3.745 | 0.2927 | Yes |
| 7 | TYMS |  |  | 829 | 3.738 | 0.3433 | Yes |
| 8 | CHAF1A |  |  | 971 | 3.525 | 0.3808 | Yes |
| 9 | PSMD14 |  |  | 1069 | 3.392 | 0.4198 | Yes |
| 10 | CCT4 |  |  | 1148 | 3.309 | 0.4592 | Yes |
| 11 | PRKDC |  |  | 1234 | 3.204 | 0.4965 | Yes |
| 12 | FKBP1A |  |  | 1267 | 3.170 | 0.5374 | Yes |
| 13 | UBE2V2 |  |  | 1406 | 3.037 | 0.5685 | Yes |
| 14 | MTIF2 |  |  | 1548 | 2.905 | 0.5975 | Yes |
| 15 | DFFA |  |  | 1721 | 2.748 | 0.6221 | Yes |
| 16 | SUMO1 |  |  | 1736 | 2.736 | 0.6584 | Yes |
| 17 | SNRPC |  |  | 1939 | 2.581 | 0.6785 | Yes |
| 18 | FNTA |  |  | 3121 | 1.878 | 0.6153 | No |
| 19 | CDC6 |  |  | 3185 | 1.847 | 0.6357 | No |
| 20 | CSNK2B |  |  | 4051 | 1.492 | 0.5910 | No |
| 21 | POLD2 |  |  | 5531 | 0.964 | 0.4929 | No |
| 22 | ACO2 |  |  | 5713 | 0.907 | 0.4917 | No |
| 23 | PRIM1 |  |  | 6525 | 0.642 | 0.4394 | No |
| 24 | GGT1 |  |  | 7852 | 0.166 | 0.3419 | No |
| 25 | EIF2S1 |  |  | 8146 | 0.058 | 0.3207 | No |
| 26 | MAZ |  |  | 9415 | -0.521 | 0.2324 | No |
| 27 | C1D |  |  | 9559 | -0.594 | 0.2297 | No |
| 28 | EWSR1 |  |  | 9674 | -0.657 | 0.2301 | No |
| 29 | EIF2B1 |  |  | 10686 | -1.309 | 0.1719 | No |
| 30 | TMEM131 |  |  | 11385 | -1.917 | 0.1456 | No |
Table: GSEA details [plain text format]

  

Fig 2: ZHAN\_MULTIPLE\_MYELOMA\_SUBCLASSES\_DIFF: Random ES distribution      
 Gene set null distribution of ES for **ZHAN\_MULTIPLE\_MYELOMA\_SUBCLASSES\_DIFF**
